# Interventions to support adolescents and young adults with the healthcare transition from paediatric to adult nephrology health services: A scoping review protocol

**DOI:** 10.12688/hrbopenres.13684.2

**Published:** 2023-07-27

**Authors:** Melissa Kinch, Thilo Kroll, Diarmuid Stokes, Suja Somanadhan

**Affiliations:** 1 School of Nursing, Midwifery and Health Systems, University College Dublin, Belfield, Dublin 4, Ireland

**Keywords:** Kidney Disease, Renal Disorder, Rare Renal Disorders, Transition Care, Transitional Care, Transfer to Adult Care, Young Person

## Abstract

**Background:** Due to technological advancements and improved medical management of adolescents and young adults (AYAs) living with renal disease, there has been an exponential increase noted in the number of patients advancing from the paediatric to adult nephrology healthcare setting. Subsequently, more AYAs are required to undergo the process of healthcare transition from paediatric to adult healthcare services. This process is often a challenging period for young people and families and is often associated with a decline in physical and psychosocial health outcomes of AYAs with renal disorders. To ensure a successful transition, AYAs must develop the ability to manage their renal condition, including the medical and psychosocial aspects of their condition, independently. Despite significant research into the transition from paediatric to adult healthcare for this unique patient cohort, the transition period remains a challenge. The scoping review will aim to map, explore, and understand the interventions that are currently available to offer positive perceptions and experiences of transition for both AYAs living with renal disorders and their families.

**Methods:** A systematic literature search will be conducted of PubMed, PsycInfo, CINAHL, ASSIA, EMBASE and Web of Science databases from the year 2000 to present. Two independent reviewers will screen the peer-reviewed literature obtained and assess them against the inclusion criteria to determine their inclusion eligibility. Data will be extracted and synthesised using a template refined by the authors. The scoping review will be undertaken in accordance with PRISMA-ScR guidelines. Data will undergo a formal critical appraisal using recognised appraisal tools.

**Conclusions:** Through mapping this knowledge, the scoping review will aim to identify interventions that are currently available and identify gaps within the literature. This evidence may support the development of transitional care interventions in the future, promote patient satisfaction, and improve patient outcome measures and experiences.

## Introduction

The number of young people advancing from paediatric to adult nephrology care has increased dramatically in recent years; patient survival rates have increased by 85–90% ( Ferris *et al*., 2006; Lewis *et al.,* 2009; Watson *et al.,* 2011). Over the past few decades, earlier diagnosis, and significant medical and technological advancements have accelerated patient survival rates and improved life expectancy ( Joly *et al.*, 2015; Stepien *et al.,* 2021). Hence, more patients are required to undergo healthcare transition, generating an increased need for a structured, well-planned transition from the paediatric to adult healthcare setting ( Joly *et al.*, 2015).

Transitional care was first described in the literature approximately 40 years ago and is now considered to be crucial in the context of adolescent health care ( Barbero, 1982; Wildes *et al.*, 2023). Transition is defined as *‘the purposeful, planned movement of adolescents and young adults with chronic physical and medical conditions from child-centered to adult-oriented health-care systems’.* ( Blum *et al.,* 1993, p. 570). Blum *et al.* (1993), p. 573, further defined how transition *‘attends to the medical, psychosocial, and educational/vocational needs of adolescents’.* Transitional care aims to maximise lifelong functioning and patient potential by providing high-quality, continuous and developmentally appropriate healthcare to adolescents and young adults (AYAs) and families when moving between paediatric and adult health services ( American Academy of Pediatrics *et al.*, 2002). It is essential that transitional care is carefully differentiated from the “transfer” of care; “Transition” to adult healthcare refers to the process that commences during childhood and ends once the patient is fully integrated, independent, and comfortable in the adult healthcare system, whereas the “transfer” of care is the distinct point where the patient moves to the new healthcare setting or provider, and is just one aspect of the multi-faceted transition process, which must be anticipated by AYAs and families and well-co-ordinated ( Marks, 2022).

There are various interventions available to support the transition process for AYAs and families living with chronic conditions. These interventions include summer camps, clinics, guidelines, transition readiness scales, online educational and interpersonal communication programmes, e-health *i.e.*, the use of mobile apps, the use of digital monitoring devices, problem solving challenges, role-play, arts-based interventions *etc*. ( Callinan & Coyne, 2020; Catarino *et al.*, 2021; Miller, 2022). The purpose of this scoping review is to map out the interventions that are available to support transition for AYAs living with renal disorders, specifically. Transition is particularly challenging for AYAs living with renal disorders due to the complexity of their conditions. For example, patients with renal disorders may receive renal transplantation or other specialist treatments, such as enzyme replacement therapy for Fabry’s disease ( Quaglia *et al.*, 2014; Riccio *et al.*, 2020).

Furthermore, moving from paediatric to adult care occurs during an emotionally vulnerable phase in the young person’s life ( Bell, 2022). The transition process occurs in tandem with the necessary life transition from adolescence to adulthood, which is well-recognised as a period of rebellion, engagement in risk-taking and impulsive behaviour, and non-adherence attributed to ongoing and incomplete development and maturation; a period where adolescents often experiment and feel unconquerable ( Foster, 2015; Kreuzer *et al.,* 2019; Tareen & Tareen, 2017; Watson, 2005). This adolescence/young adulthood period occurs between the ages of 14–24 years, in the context of brain development and maturation ( Watson *et al.,* 2011). Hence, young people in this age bracket must be carefully considered when planning transition ( Bell, 2007; Watson *et al*., 2011).

The role of the family in the transition process must also be recognised and considered. As discussed by Betz, Nehring and Lobo (2015), the healthcare transition process instigates a realignment of the relationship between AYAs and their parents or caregivers. AYAs must learn how to self-manage their condition, a role which may have previously been managed by their parents or caregivers. Further, parents or caregivers themselves must be prepared for the transition process. A study undertaken by Shaw, Baldwin and Heath (2021) identified that parents/caregivers struggle to let go of their current role in their young person’s healthcare due to feelings of fear or uncertainty regarding their AYAs future once they reach adulthood. Families must receive adequate support and guidance during the transition process, and their role must not be forgotten. Though this scoping review will specifically search for interventions to support a positive transition process for AYAs with renal disorders, interventions that also refer to the role of the family in the process will be included, as the role of the family is integral to a successful transition of care.

Despite the complexity of the transition process, evidence suggests that most AYAs and families receive inadequate or a total absence of transition preparation ( Gabriel *et al.,* 2017). If AYAs transition to adult healthcare in a scenario whereby they are not able to manage their disease adequately, undesirable health outcomes may arise ( Ma *et al.,* 2021; Young *et al.,* 2010). This is problematic and adds to the already challenging period that AYAs face at this time; AYAs are navigating their adolescent development transition period, whilst also learning to manage the various aspects of their healthcare, grieve losses in their lives, and attend to the practicalities of various treatments, interventions, and appointments that they undergo ( Kralik & Van Loon, 2010). In addition to this challenging developmental period, young people with kidney disease have specific medical needs that they must manage, such as steroids, which often alter their appearance and may affect their mood, their growth may be stunted, they may be faced with extreme lifestyle modifications inclusive of fluid and diet restrictions, intensive medication regimens with side effects, and AYA’s may have to undergo dialysis, which comes with side-effects such as fatigue, and is time consuming and restricts their activity ( Dallimore *et al.*, 2018).

A failure to transfer AYAs using carefully planned, well-designed transition programmes with the required competencies to manage their condition, may lead to poor attendance at adult nephrology units ( Çiçek & Alpay, 2022). In turn, AYAs are placed at high risk of clinical deterioration, resulting in a cascade of negative health outcomes ( Foster & Bell, 2015; Kreuzer *et al.,* 2019; Prüfe *et al.*, 2022; Watson *et al.,* 2011). This may be attributed to multiple factors and not exclusively poor attendance to adult clinics, such as non-adherence to medications or medical treatment and regimens, including non-adherence to life-long immunosuppressive regimens, leading to an increased risk of rejection and allograft loss and oftentimes a return to dialysis ( Akchurin *et al.,* 2014; Campbell *et al.,* 2016; Crawford *et al.,* 2020; Dobbels *et al.,* 2010; Dobbels *et al.,* 2009; Foster, 2015; Foster & Bell, 2015; Kreuzer *et al.,* 2019; Mazzucato *et al.,* 2018; Prüfe *et al*., 2022; Quaglia *et al.,* 2014; Wildes *et al.,* 2023).

Moreover, the transition process must be considered for AYAs living with rare renal disorders. Transition is often challenging for AYAs and families living with rare renal disorders. Over 300 inherited, congenital, and acquired renal disorders meet the epidemiological criteria to be defined as rare renal disorders ( Bassanese *et al.,* 2021). The list of rare renal disorders is constantly growing ( Joly, *et al.*, 2015). In Europe, renal disorders have an estimated prevalence of approximately 60–80 cases per 100,000, with nearly all children who progress to renal-replacement therapy living with an inherited kidney disease ( Devuyst *et al.,* 2014). Moreover, children born with rare renal disorders, particularly severe congenital nephropathies, and their families often live with impaired physical, cognitive, and psychosocial development ( Aymé *et al.,* 2017; Devuyst *et al.,* 2014). Many young people living with such conditions are now surviving into adulthood with conditions that were previously unknown to or not frequently seen by adult nephrologists ( Marks, 2022).

Hence, adult nephrology services are receiving a growing number of AYAs with rare renal disorders who have transitioned from paediatric services ( Watson *et al.,* 2011). The transition from the paediatric to the adult healthcare setting for these patients has been reported to be a challenge to transfer, due to a lack of training and knowledge of adult nephrologists of these rare conditions, amongst several other reasons ( Prüfe *et al.,* 2017; Watson *et al.,* 2011). However, this cohort of patients must be carefully considered, as congenital anomalies of the kidney and urinary tract have been cited as the most common causes of end-stage and chronic kidney disease in childhood ( Marks, 2022). These patients and their families must be carefully transitioned from paediatric to adult health care, with emphasis placed on the need for these patients and families to have adequate knowledge surrounding how to manage their health and transfer to adult-centred care with up-to-date medical knowledge ( Gabriel *et al.,* 2017).

Transition aims to gradually prepare AYAs and families for a successful integration into the adult healthcare system in a stepped, co-ordinated and uninterrupted manner ( Matsuda-Abedini *et al.*, 2023). A positive transition begins in the paediatric health setting and prepares the adolescent to become a self-responsible adult patient who manages their condition appropriately and ends with the patient finding their place in the adult healthcare system in a safe and secure manner ( Kreuzer *et al.,* 2019; Paone *et al*., 2006). Transition is considered successful if the patients’ health competence, psychosocial needs, and self-determination are promoted, and when the patient has improved decision-making abilities and the ability to communicate their care effectively ( Kreuzer *et al*., 2019; Prüfe *et al*., 2022; Watson *et al.,* 2011).

The importance of an effective transition programme, incorporating well-planned interventions and strategies for young people living with renal disorders is apparent. Studies have demonstrated that the highest rate of kidney transplant loss occurs during the transition years and immediately following transfer of care ( Foster, 2015; Foster & Bell, 2015; Kreuzer *et al.,* 2019; Prüfe *et al.*, 2022; Watson *et al.,* 2011). Therefore, this age group must be carefully managed, as graft failure often requires a return to dialysis, reducing the patient’s quality of life, increasing morbidity and mortality rates, shortening AYAs life expectancy, and heightening health care expenses ( Prüfe *et al*., 2022). Providing increased knowledge, awareness, and education to AYAs surrounding their condition and the transition process, and providing appropriate, relevant, easily accessible self-management strategies may better support AYAs with the transition process. Improved psychosocial support, the management of potential extrarenal complications, and the provision of genetic and reproductive counselling are other integral components that must be integrated within the transition programme ( Aymé *et al.,* 2017).

Moreover, it has been established within the literature that the transition process should start early, by at least 12 to 14 years old, providing sufficient time to prepare patients and parents/caregivers for the transition of care ( Aymé *et al.,* 2017; Willis & McDonagh, 2018). Nonetheless, the timing of transfer must be individualised, and healthcare providers must be cognisant of patient-specific factors including growth, maturity, and overall readiness to transition ( Aymé *et al*., 2017). The provision of an effective transition programme may reduce the risk of declining renal function and acute kidney rejection episodes and may also improve long-term graft outcomes in AYAs ( Raina *et al*., 2018). Due to the complexity of renal diseases, transition support and services must be carefully planned and timed, and focus on empowering a safe transfer of care from the child to the adult healthcare setting for AYAs and families living with renal diseases, to ensure the risk of negative health outcomes is mitigated.

A preliminary search of Medline, PROSPERO ( PROSPERO, 2022), the Cochrane Database of Systematic Reviews, and Google was conducted on the 1 ^st ^of October 2022. An ongoing systematic narrative review protocol was located on PROSPERO by Piccoli *et al.* (2020), which will explore the best approach for the clinical management of young people with chronic kidney disease undergoing transition. However, this scoping review differs in that it aims to scope the literature and map any intervention to support transitional care for AYAs with renal disorders within healthcare settings, inclusive of rare renal disorders, chronic kidney disease, patients undergoing dialysis, and transplant recipients. For this review, an intervention may be defined as any programme, service, intervention, clinic, scale, model, tool, or activity that aims to support AYAs with renal disorders as they transition from paediatric to adult healthcare services. 

## Methods

The methodology for this review employs the five-stage framework, as outlined by Arksey and O’Malley (2005), incorporating more recent methodology refinements as proposed by Levac, Colquhoun and O’Brien (2010) and the Joanna Briggs Institute (JBI) ( Peters *et al*., 2015; Peters *et al.,* 2020). Arksey and O’Malley (2005) also identified an optional ‘consultation exercise’ to inform and validate findings, as a sixth stage at the end of the review ( Arksey & O’Malley, 2005).

This step has been identified as a stage that will improve the review remarkably ( Arksey & O’Malley, 2005). Researchers from various academic and health disciplines who have conducted research or cared for patients and families undergoing healthcare transition will be invited to be a part of the scoping review, and appraise and validate the review findings. The Preferred Reporting Items for Systematic reviews and Meta Analyses Scoping Review extension (PRISMA-ScR) checklist will be utilised to increase methodological transparency and ensure consistency when publishing findings ( Tricco *et al.,* 2018). The six-stage scoping review framework developed by Arksey and O’Malley (2005), incorporating guidance from the JBI ( Peters *et al.*, 2020), will be employed to guide the scoping review.

### Aims/objectives

The objective of this scoping review is to map the various interventions that are available within the literature to support AYAs living with renal disorders as they transition from the paediatric to the adult healthcare system. The following research questions will be addressed:

1. Identify, appraise, and synthesise knowledge surrounding the interventions available to support a successful healthcare transition for young people living with renal disorders.
2. Ascertain whether the AYAs were involved in the intervention’s development.
3. Determine the study settings and geographical contexts, and the study types conducted ( *i.e.* qualitative, quantitative or mixed-methods).
4. To understand the experiences, barriers and facilitators recognised by young people living with renal disorders, of current transition interventions.
5. Identify research and knowledge gaps in the literature.

### Stage one: Identification of the scoping review research question

The scoping review question guides and directs the development of the review process. In this review, the population, concept, and context (PCC) framework will be utilised, which has been recognised as an effective framework for a scoping review by the JBI ( Peters *et al.,* 2020). The preliminary question is:

What interventions are available to support the transition of care (Concept) from the paediatric to adult healthcare setting (Context) for adolescents and young adults living with renal disorders (Population)?

### Stage two: Identifying relevant studies

A systematic literature review aims to obtain as many relevant studies as possible on a particular topic, as opposed to every study available using a thorough, objective, and reproducible search ( Levay & Craven, 2019). Being systematic minimises bias ( Levay & Craven, 2019). The review question has been formulated using the PCC framework. This ensures that appropriate, relevant search terms can be developed, ensuring the retrieval of pertinent results on the interventions available to support the transitional care of young people living with renal conditions, and will ensure that the retrieval of irrelevant results is minimised.

***Eligibility criteria***

The inclusion and exclusion criteria is developed through the generation of a PCC table ( Table 1), as recognised by Boland *et al.* (2017) as an effective means of defining criteria. This process remains iterative, and any changes made throughout the review will be recorded and documented to maintain transparency and rigor in reporting.

**Table 1.** Inclusion and exclusion criteria.

**Review question**	What interventions are available to support the transition of care from the paediatric to adult healthcare setting for adolescents and young adults (AYAs) living with renal disorders?	
	**Inclusion**	**Exclusion**
**Population**	Humans. Studies that include AYAs (12–24 years old), who are living with a renal disorder. AYAs must be: (a) preparing for transition; (b) currently undergoing transition; (c) having undergone transition from the paediatric to the adult healthcare setting. Studies that refer to families, caregivers, healthcare providers, programme managers and policymakers involved in the transition process alongside the AYAs. Male, female and non-binary individuals.	Any study population other than humans, *i.e.*, animals. Studies that do not include AYAs between 12–24 years old who are living with renal disorders. Studies that do not refer to AYAs undergoing the transition process from the paediatric to the adult healthcare setting.
**Concept**	The concept of transition from the child-centred to the adult centred healthcare system. Transitional care interventions – any intervention, for example, programme, service, intervention, clinic, or activity that aims to support the movement of AYAs and families from paediatric to adult healthcare services.	Does not refer to transition from a child-centred to adult-centred healthcare system. Any intervention that is not designed to support transition.
**Context**	AYAs with renal disorders, including chronic renal disorders, rare renal disorders, kidney transplant recipients and AYAs on renal dialysis.	Studies that do not refer to renal disorders, including chronic renal disorders, rare renal disorders, kidney transplant recipients and AYAs on renal dialysis.
**Study design and ** **study focus**	All primary peer-reviewed studies of any study design.	Studies that are not primary peer-reviewed studies.
**Setting/geographical** ** location**	Health services, including hospitals (various areas within the hospital such as wards, outpatient departments etc.). Interventions provided in the community by healthcare professionals from secondary or tertiary health services will also be included. No limits will be placed on geographical location, both national and international literature. No limits will be placed on language.	Studies that refer to any other transition process, other than the transition process from the paediatric to adult healthcare services. Studies that do not refer to secondary or tertiary healthcare services. Other health service areas such as respite or residential centres, or special care.
**Time period**	Studies on or after the year 2000 to current.	Any study prior to the year 2000.

***Population***

This scoping review will include AYAs living with any renal condition, encompassing any chronic or rare renal condition, patients who are currently on renal dialysis, and renal transplant recipients. Though this scoping review will specifically search for interventions to support AYAs with the healthcare transition, studies that refer to families, caregivers, healthcare providers, programme managers and policymakers involved in the transition process alongside the AYA will also be included, given the important and invaluable role that they play in the transition process.

**Age**

Studies will be included if they refer to AYAs between the ages of 12–24 years old, who are either (a) preparing for the transition process, (b) currently undergoing the transition process, or (c) having undergone transition from the paediatric to the adult healthcare setting. This age range is deemed appropriate, as 13/14 years old is the recommended age to commence the transition process, and through the inclusion of patients up until the age of 24 years, we can retrieve results from patients who may have completed the process ( Willis & McDonagh, 2018). Furthermore, this age group is appropriate, as the International Society of Nephrology and the International Pediatric Nephrology Association have recommended that transition should occur between 14 and 24 years old ( Joly *et al.*, 2015). By including 12 and 13 year olds, we can capture patients who are about to commence transition.

**Gender**

Studies that refer to male, female and non-binary young people with renal disorders will be included in the review. This will avoid gender bias ( Upchurch, 2020). Gender is often characterised as female, male or non-binary. This review will include all genders to promote inclusivity and to widen participation.

***Concept***

This review will examine the concept of transition, which may be defined as the purposeful, planned process whereby adolescents move from paediatric, family-centred health services to adult, person-centred health services ( Campbell *et al.,* 2016). This review will examine interventions that support the transition process. For this review, interventions encompass any programme, service, intervention, clinic, or activity that aims to support the movement of AYAs and families from paediatric to adult healthcare services.

***Context***

**Setting**

This scoping review will consider primary research studies that have been conducted in multiple different settings within secondary and tertiary level health services, including hospitals (including various areas within the hospital such as wards or outpatient departments). Nonetheless, studies that refer to interventions provided in a community setting by healthcare providers from secondary or tertiary healthcare services will be included. This decision has been made, as this scoping review is part of a larger PhD study examining healthcare transition in secondary or tertiary healthcare settings. The results of this review will feed into the primary research study which focuses on how healthcare professionals from secondary or tertiary health services can support young people and families living with renal disorders with healthcare transition.

**Geographical location**

No limits will be placed on the geographical locations included within this study. Furthermore, there will be no language limits placed on our initial search. This ensures that language and publication bias is avoided. As discussed by Boland *et al.* (2017), language bias may arise if only English-speaking countries are included due to English-language journals being more likely to publish positive results. Due to resource limitations, the literature will be searched in English, with no restrictions placed on language. This ensures maximum retrieval of literature in all potential languages. Efforts will be made to translate study abstracts, where possible, using translation tools such as google translation services. However, literature that is obtained but not in the language of choice (English), and that cannot be readily translated due to resource limitations, will be excluded ( Gough *et al.*, 2017).

Nonetheless, by not setting language limitations when searching, all potential literature can be obtained and made readily available for other researchers who may have the resources to translate the articles that are obtained ( Gough *et al.*, 2017).

***Types of sources***

This scoping review will consider all primary peer-reviewed study designs that explore interventions to support the transition process amongst young people with renal disorders, including quantitative, qualitative or mixed-method study designs or methodologies. This will ensure that the authors can capture studies which refer to interventions and also those that refer to AYAs/families/healthcare professionals experiences of those interventions, where relevant. In the initial reviewing process, both studies available in full-text and abstract format will be considered for review. This will ensure that the most up-to-date evidence is not missed ( Boland *et al*., 2017). Any literature that does not present data meeting the inclusion criteria will be excluded.

Only peer-reviewed papers that have been published within academic journals will be included in this scoping review. Literature reviews, case studies, individual case reports, opinion pieces, commentaries, editorials, and conference abstracts will be excluded from the review, based on resource availability to the primary researcher (MK), including a limited amount of time and funding to undertake the review as part of an academic degree. Furthermore, the inclusion of primary, peer-reviewed studies has been selected, as the authors seek to understand how the interventions identified were developed. Developing this understanding of the theoretical and methodological underpinning of the intervention’s development will support the authors in the next phase of the research study. Nonetheless, the authors recognise the importance of including grey literature in scoping reviews. Given that the authors primary PhD study will be conducted in the context of the Irish healthcare setting, national grey literature will be sourced and included in the discussion of the scoping review, to compare the international interventions sourced with those used within the Irish healthcare setting.

***Search strategy***

The formulation of a search strategy is invaluable and must be transparent, accountable, and replicable, ensuring a systematic process is followed ( Gough *et al*., 2017). A comprehensive search strategy was developed by the primary researcher in consultation with an expert librarian (DS) with experience in undertaking literature searches and using thesaurus tools ( Table 2). To develop the search strategy, an initial scoping search was conducted in the databases CINAHL Plus with Full Text, PubMed, Applied Social Sciences Index and Abstracts, PsycINFO, Web of Science and Embase on the 2 ^nd^ March 2023. This identified articles relevant to the topic. Free-text words and subject headings/index terms were located from titles, abstracts, and main texts of articles, and were used to further enrich and inform the search strategy ( Boland *et al*., 2017).

**Table 2.** Search strategy.

Research question themes	Search terms
Population	“young people” OR youth* OR adolescen* OR teen* OR “young person*” OR juvenile* OR child* OR kid OR kids OR minor* OR pre-adult* OR preadult* OR “pre adult*” OR “young adult*” OR child-hood OR pubescen* OR puberty OR “emerging adult*” OR pediatric* OR paediatric*
Concept	intervention* OR program* OR tool* OR model* OR scale* OR framework* OR clinic* OR e-health* OR mHealth* OR eHealth* OR m-health* OR “mobile health*” OR “electronic health*” OR “digital health*” OR digi* OR digi-health OR procedure* OR protocol* OR pathway* OR manual* OR strateg* OR measure* OR application* OR app OR apps OR “mobile application*” OR “mobile health application*” OR workshop* OR recommendation* OR support* OR instrument* OR website* OR web OR guide* OR guidance OR activit* OR mechanism* OR approach* OR method* OR scheme* OR digital-health* OR software* OR telemedicine OR “tele medicine” OR tele-medicine OR telenursing OR “tele nursing” OR tele-nursing OR telecare OR tele-care OR “tele care” OR telehealth* OR tele-health* OR “tele health*” OR policy OR policies OR resource* OR initiative* OR curriculum* **AND** transition* OR “transfer to adult care” OR “transfer from pediatric to adult care” OR “transfer from paediatric to adult care” OR “transfer to adult health*” OR “transfer care” OR “care transfer*” OR “healthcare transfer*” OR “health-care transfer*” OR “health transfer*” OR “medical transfer*” OR “patient transfer*” OR “care coordination*” OR “care co- ordination*” OR “co-ordination of care” OR “coordination of care” OR “care continuum*” OR “continuum of care” OR “patient handoff*” OR “clinical handover*” OR “continuity of care” OR “continuity of patient care” OR “care continuity” OR “healthcare continuity” OR “health-care continuity” **AND** “healthcare setting*” OR “health care setting*” OR “health-care setting*” OR “health care facilit*” OR “health-care facilit*” OR “health care service*” OR “healthcare service*” OR “health service*” OR “health-care service*” OR hospital* OR clinic* OR outpatient* OR “emergency department*” OR “emergency room*” OR “emergency service*” OR “emergency medical service*” OR ward* OR inpatient* OR “tertiary health*” OR “tertiary care” OR “tertiary referral cent*” OR ED OR ER OR “A&E” OR in-patient* OR out-patient* OR “secondary health*” OR “secondary care” OR “secondary referral cent*” OR “ambulatory care” OR “ambulatory health*” OR “acute care” OR “acute service*” OR “acute health*” OR “emergency care” OR “accident and emergency” OR “health facilit*” OR “healthcare facilit*” OR “paediatric unit*” OR “pediatric unit*” OR “care unit*” OR ICU OR PICU
Context	renal OR kidney* OR nephrol* OR dialysis OR haemodialysis OR hemodialysis OR allograft* OR graft* OR transplant* OR “renal replacement therapy” OR “kidney replacement therapy”

Thesaurus tools, subject headings and synonyms were utilised and shared across databases to maximise search results. Furthermore, the use of truncation was utilised ( Gough *et al*., 2017). The search strategy encompasses the use of Boolean operators, subject headings and free-text headings for each database. The librarian (DS) will provide support and assistance to the primary researcher when carrying out searches of the databases.

Furthermore, the literature search strategy utilised will be reported in the scoping review, within a table in the appendix, to ensure easy replication by others ( Tricco *et al.,* 2018). The search strategy is iterative ( Gough *et al*., 2017). Therefore, any modifications or adjustments to the search strategy as the review progresses, will be clearly documented to ensure transparency in searching. Databases will be limited to the year 2000, ensuring that the results portray relevant interventions that consider medical and technological advancements within the realm of healthcare over the past two decades. Furthermore, only peer-reviewed literature will be included in the scoping review.

To ensure a comprehensive mapping of the literature, a broad range of databases will be searched. The databases that will be searched include: (1) health databases, including PubMed, CINAHL Plus with Full Text and Embase; (2) social sciences databases, including PsycInfo and ASSIA; and (3) multidisciplinary databases, including Web of Science.

The rationale for searching these particular databases is justified and will ensure that the results portray the breadth of evidence available. Furthermore, the databases selected encompass health, social sciences and multidisciplinary databases. These databases have been chosen strategically, as transitional care requires a multi-disciplinary approach, and must include not only physical support, but also psychosocial support ( Aymé *et al.,* 2017). Therefore, it is essential to not only include health databases, but also social sciences and multidisciplinary databases. The author will not undertake complementary search techniques, including bibliographic searching and hand searching, in addition to searching databases due to the time restrictions of the primary author to complete the review in a timely manner as a component of their academic degree.

### Stage three: Study selection

Each search will be documented systematically, including the date, search terms, and the results per search string. Following the literature search, all results obtained through the databases will be collated and imported into a bibliographic reference manager EndNote 20. All imported results will be dated and saved to individual libraries, depending upon which database they were collected from. This enables the authors to keep a record and manage their search results. Following the import of the results to EndNote 20, all duplicates will be removed.

**Figure 1.**
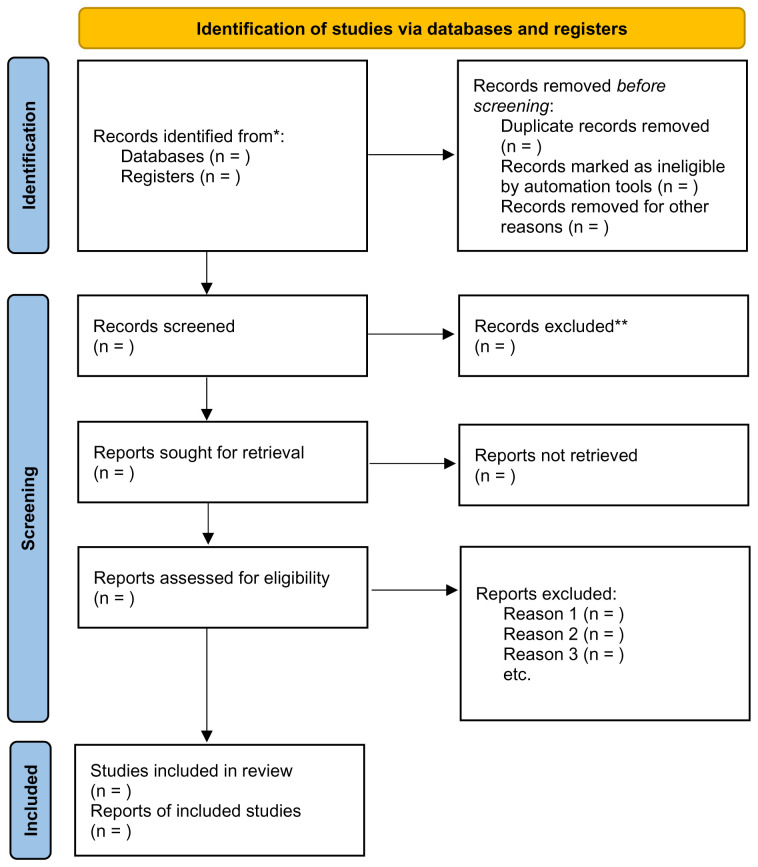
PRISMA 2020 Flowchart for Scoping Review ( Page *et al.*, 2021).

The number of articles retrieved following these steps will be added to the PRISMA-ScR flowchart, as recommended by the JBI ( Peters *et al.,* 2020) ( Figure 1). Furthermore, a PRISMA-ScR flowchart will be completed following the completion of each search to ensure transparency when mapping the number of records identified and to provide a rationale as to why studies were excluded ( Page *et al.,* 2021).

Search results from EndNote will be imported for further screening and reviewing in Covidence systematic review screening and data extraction software tool ( Covidence, 2022). Once complete, the screening and data extraction process can commence. This process will be undertaken in three steps: (1) title and abstract screening; (2) full-text review; (3) data extraction in Covidence. Following the removal of duplicates, screening will be performed. The titles and abstracts of all studies obtained will be screened against the inclusion and exclusion criteria ( Boland *et al.,* 2017). Two reviewers will screen all results independently (at both title and abstract and full text stage), before being included or excluded in the review. This reduces bias and makes the review process more robust ( Boland *et al*., 2017).

A pilot testing of the literature obtained (n=100) will be undertaken using Covidence software with the inclusion and exclusion criteria inputted to the software tool at both screening stages to ensure that all reviewers are satisfied with the criteria, and so as relevant amendments can be made if required. Undertaking a pilot test of the screening process is recommended by Peters *et al.* (2020). This process will be transparent, and document all decisions made surrounding which literature was included or excluded and a rationale for inclusion or exclusion. Covidence will support this process by tracking/logging decisions made throughout the process. Any discrepancies or lack of consensus between authors during the screening process will result in the second author (TK) being consulted and adjudicating. Furthermore, if any missing data is noted within papers, the authors will be contacted by the primary author (MK). This will ensure maximum retrieval of full-text papers when there is no full report available online.

### Stage four: Data charting

In scoping reviews, data extraction is often referred to as “data charting” ( JBI, 2022). Data charting will be undertaken using a version of the JBI data extraction tool, referred to as a charting table, modified by the authors to fit the objectives of the scoping review ( Table 3) ( Peters *et al.,* 2020). An example of the data charting tool will be included in the appendix. This charting form will be piloted on two articles by two independent authors, on articles chosen at random.

**Table 3.** Sample data charting form.

Data chart heading	Description
Author(s)/Year	Name of author(s)/The year that the article was published
Title of article	Title of the article or study
Origin/Country of Origin	Where the article/study was published/conducted
Study Aims/Purpose	The aims of the study
Population and sample size	Population/sample size within evidence
Research Design/ Methodology	Research methods/design/techniques employed
Quality Appraisal	Critical appraisal through appraisal tools
Data Analysis	Analysis of the data
Study setting	The setting the study was conducted in
Intervention type	Details surrounding the intervention and its duration (if applicable)
Co-Design	Were AYAs/Families involved in the interventions design?
Experiences	What was the experience of the AYAs/families surrounding the named intervention? What were the barriers/enablers of the named intervention?
Findings/Conclusion	Important findings from the research, including strengths and limitations of the study/intervention, areas for future research/ gaps in the knowledge and any important aspects of the conclusion

This tool may be further refined in the subsequent review during the data charting process, as the review progresses. Modifications will be detailed in the scoping review for transparency in reporting. The data that will be charted includes details surrounding the population, concept, context, study methodology, quality appraisal, and findings that are significant to the review’s aims and objectives. We will also chart information on the interventions including their use and whether the intervention was co-designed with AYAs and families. The preliminary data charting process will be undertaken by the primary author (MK) and will be audited and reviewed by the fourth author (SS). Once both the first and fourth author reach consensus on the data charting table, it will be made available for all authors involved in the review through the utilisation of a shared Google drive. The table will be discussed with all other reviewers to seek their feedback and make further refinements, as necessary until all authors reach consensus.

***Quality appraisal***

Though optional, for the scoping review a quality appraisal will be undertaken, using the Mixed Methods Appraisal Tool (MMAT) ( Hong *et al.,* 2018) and the Critical Appraisal Skills Programme (CASP) ( Critical Appraisal Skills Programme, 2022). Quality appraisal or critical appraisal is defined as a process whereby research is evaluated carefully and systematically in terms of the trustworthiness, relevance, and value of the research ( Burls, 2014). This process has been identified as a process that will improve the quality of the study through careful evaluation of the research studies retrieved in terms of their methodological quality.

### Stage five: Collating, summarising and reporting results

The results of the literature search and screening process will be presented within a PRIMSA-ScR flow diagram ( Peters *et al.,* 2020). Key constructs will be summarised through descriptive content analysis by the authors, in line with the JBI guidance, which advocates that scoping reviews should only incorporate basic descriptive analysis ( Peters *et al.,* 2020).

Quantitative studies retrieved will be analysed using basic frequency counts of concepts, populations, and study locations, followed by a narrative summary ( Peters *et al.,* 2020). The purpose for providing frequency accounts of study locations, concepts and populations is to enable the author to map the geographical areas and populations that are most represented in the current literature. This will further identify gaps in the literature and prioritise areas for future research. Qualitative studies will be summarised through descriptive content analysis by the authors ( Peters *et al.,* 2020). Basic data coding data will be undertaken by the authors to categorise data and enable identification and clarification of concepts or definitions within the field ( Peters *et al.,* 2020).

The "PAGER" framework will be utilised as a framework to summarise and report the results of the review. This tool provides a standardised approach to analysing, reporting and synthesising scoping review findings, and is based on ‘patterns’, ‘advances’, ‘gaps’, ‘evidence for practice’ and ‘research recommendations’ found within the literature ( Bradbury-Jones *et al.,* 2022). Recommendations for future research will be presented relative to the scoping review findings. The results of the review will be discussed between the authors to further enhance, validate and add meaning to the results obtained. The results of the review will be presented in the form of diagrams, tables, graphs and a narrative summary of the findings. The authors will be transparent and explicit in their approach to data analysis, summarising and reporting the scoping review results, and will document all decisions made throughout the review, as recommended by Peters *et al.* (2020).

### Stage six: Consultation

Consultation for this scoping review will be undertaken in the form of an expert reference panel that will be invited to discuss the reviews findings and the implications for practice. Reviewers will consult with and invite experts from Universities and Healthcare Settings in both Northern Ireland and the Republic of Ireland to act as an expert panel for this review. It is anticipated that this panel will include individuals with expertise in transitional care, paediatric nephrology multidisciplinary teams, and staff members from the world of academia. Furthermore, and importantly, the review results will be distributed to public and patient involvement (PPI) groups, including AYAs with renal disorders and families who will be invited to provide feedback on the results of the review. This review is part of a larger PhD study, which is being undertaken by the primary reviewer (MK), and the scoping review will feed into the design of the primary research study.

### Dissemination plans for completed scoping review

Various dissemination strategies will be employed following the completion of this scoping review. The results of the scoping review will be shared amongst various academic institutions both within the Republic of Ireland and Northern Ireland. The results will be shared with multi-disciplinary teams within both adult and paediatric clinical settings on a national level that care for AYAs with renal disorders undergoing transition. The results will also be shared with PPI groups, including young people and families living with renal disorders that have experienced the healthcare transition process. The authors will engage with these teams and population groups to share and discuss the review findings and interpretations and to gather their perspective on the findings obtained from the review. The primary author will also strive to present the findings of this scoping review, through oral and poster presentations, at local, national, and international conferences, as can be facilitated. Finally, post completion of the scoping review, the author will seek publication in a peer reviewed journal, such as the Journal of Pediatric Nursing. Publication in a peer-reviewed academic journal is an excellent means of reaching and sharing findings with relevant academic, clinical and research audiences ( Boland *et al*., 2017).

## Study status

 This scoping review is currently at stage four: data charting. Database searches and screening have been completed.

## Conclusions

For AYAs and families living with renal disorders, the transition from paediatric to adult healthcare can be challenging. Transition is often associated with risk, health status deterioration and psychological and social stress. There is an urgent need to better understand the interventions that support AYAs living with renal disorders to obtain the required competencies to support transition to the adult healthcare setting. This scoping review will explore what is known about such interventions to promote patient satisfaction and improve patient outcome measures and experiences.

The results will synthesise the theoretical and empirical evidence available. These findings may assist with the future development of transitional care interventions for AYAs with renal disorders. This scoping review is part of a larger PhD project examining the current transition care available for AYAs and families living with renal disorders. The results will be invaluable to feed into the research project and gain a better understanding of the interventions utilised during the transition process for this cohort of patients. The findings of this review will seek publication in a peer-reviewed journal, and may be presented at local, national, and international conferences, and shared with nephrologists, the wider multidisciplinary team, researchers, AYAs and families through rare disease and renal organisations.

## Data Availability

No data are associated with this article.
