# Supplementary material for: Interventions to support adolescents and young adults with the healthcare transition from paediatric to adult nephrology health services: A scoping review protocol
Source: HRB Open Res. 2023 Oct 30;6:9. Originally published 2023 Feb 2. [Version 3] doi: 10.12688/hrbopenres.13684.3 (PMC10439360; doi:10.12688/hrbopenres.13684.3)
Supplement: Peer review history

# Reviewer report: Reviewer response for version 3

**DOI:** 10.21956/hrbopenres.15136.r36827

**Published:** 2023-11-01

**Authors:** Alison Luke

**Affiliations:** 1 The University of New Brunswick (UNB) Saint John Collaboration for Evidence-Informed Healthcare: A JBI Centre of Excellence, University of New Brunswick Saint John, Saint John, New Brunswick, Canada

I have no further comments! I approve.

Is the study design appropriate for the research question?

Yes

Is the rationale for, and objectives of, the study clearly described?

Yes

Are sufficient details of the methods provided to allow replication by others?

Partly

Are the datasets clearly presented in a useable and accessible format?

Not applicable

Reviewer Expertise:

I do research in health service delivery with a focus on models of care that support improved care integration. I have done research in the area of transition from paediatric to adult care for children and youth with complex care needs.

I confirm that I have read this submission and believe that I have an appropriate level of expertise to confirm that it is of an acceptable scientific standard.

# Reviewer report: Reviewer response for version 2

**DOI:** 10.21956/hrbopenres.15065.r35446

**Published:** 2023-08-18

**Authors:** Alison Luke

**Affiliations:** 1 The University of New Brunswick (UNB) Saint John Collaboration for Evidence-Informed Healthcare: A JBI Centre of Excellence, University of New Brunswick Saint John, Saint John, New Brunswick, Canada

Thank you for the opportunity to review the updated version of this review protocol. The protocol reads very well. I also thank the authors for addressing all of my comments. I only have a couple of minor comments/edits.

 1) On page 4 (Background) there are a few places where paragraphs start with "Moreover" (used twice) and "Hence". Typically you should not begin a paragraph with these words as they are used to build on an argument. I recommend removing these words and adjust the first sentence accordingly.

 2) Under "Objectives" you use the word "To" before understand for your forth objective. I would remove the "To" for consistency.

Is the study design appropriate for the research question?

Yes

Is the rationale for, and objectives of, the study clearly described?

Yes

Are sufficient details of the methods provided to allow replication by others?

Partly

Are the datasets clearly presented in a useable and accessible format?

Not applicable

Reviewer Expertise:

I do research in health service delivery with a focus on models of care that support improved care integration. I have done research in the area of transition from paediatric to adult care for children and youth with complex care needs.

I confirm that I have read this submission and believe that I have an appropriate level of expertise to confirm that it is of an acceptable scientific standard.

# Response

**Published:** 2023-10-25

**Authors:** Melissa Kinch

**Affiliations:** University College Dublin, Ireland

The authors would like to thank you once again for your invaluable feedback on this scoping review protocol. We have taken your feedback on board and made the relevant changes. The primary author (MK) has revised the scoping review protocol and made relevant grammatical and sentence structure amendments throughout the entire scoping review to ensure clarity.

# Reviewer report: Reviewer response for version 1

**DOI:** 10.21956/hrbopenres.14965.r34084

**Published:** 2023-06-27

**Authors:** Alison Luke

**Affiliations:** 1 The University of New Brunswick (UNB) Saint John Collaboration for Evidence-Informed Healthcare: A JBI Centre of Excellence, University of New Brunswick Saint John, Saint John, New Brunswick, Canada

Thank you for the opportunity to review this scoping review protocol. I think the review will be an important addition to the transition literature.

 Please find below some feedback and edits.

1. In the introduction, the second sentence “... *and the improved management…*” I would remove the “ *the*” and maybe add “ *…and improved disease management.*”
2. Also remove “ *the*” and “ *of patients*” in this sentence “ *…and improved life expectancy.*”
3. In the sentence “ *Hence, more patients are required to undergo the this healthcare transition.*” Please remove the “ *the*”.
4. In the methods section in the second paragraph, “ *Experts in…*” I am unclear why the author is including the specific disciplines listed. Maybe you could say that you will, include “researchers from various academic disciplines who have conducted research in this area.”
5. Under Aims and Objectives, I felt like this section was very broad. I am also unfamiliar with presenting a protocol this way (I typically follow the JBI template).

I am wondering why the authors do not mention that descriptive papers could be included as this would provide the authors with information on existing programs or interventions. If this were to be included, there would not be a “methodology”. Scoping reviews typically cast a wide net. If the goal is to map the literature on programs or interventions that support the transition from pediatric to adult healthcare for this population, the research methods or findings from a particular study could be important (would be included if present) but is it the main goal of this review? Are the authors more interested in mapping the existence of programs or are you only interested in studies that look at experiences, barriers, facilitators etc.?

 Typically, when we do scoping reviews, we are interested in mapping existing programs, but we also include (often as a secondary concept) and findings on experiences, barriers, facilitators etc). I also see that one aim is to “ *clarify the concept of transitional care.*” My concern here is that this is really beyond the scope of this review. This could be a stand alone concept analysis. Upon reading through the rest of the protocol, I do not see any mention of this and wondering what data will be extracted to address this objective. If you do want to keep it in, then you need to extract data around how authors from included articles define the concept.

1. The research question asks “ *What is the evidence base…*” I am unclear what you mean by evidence base for this context?
2. Why is grey literature being excluded? I think later on you say that it will be due to time constraints. Given scoping reviews are meant to cast a wide net, I think it is important to include grey literature if possible.
3. Under the context section, I am unclear if programs or interventions offered in community would be included? If not why? Again, if you are interested in capturing programs that support this transition, I would think you want to be broad so you do not miss any?
4. I am unclear why the authors will be doing quality appraisals. This is not typically done with scoping reviews.
5. Under study selection section, in the paragraph that starts with “ *Search results from EndNote...*” The authors provide a list for data extraction. I am wondering why each step is capitalized?
6. The screening process needs to be better clarified. The authors note that 2 reviewers will be used for initial title and abstract screening, but it also needs to be clear that there needs to be 2 screeners for the full text screening as well as the data extraction process.
7. In the paragraph that begins with “ *A pilot testing of literature obtained...*” I am unclear what is being referred to here. Please clarify.
8. In the paragraph on data charting, please include a specific list of what data will be extracted (I see this in the table, but I think needs to be here as well). In the table for data extraction, I would not have a separate category for experiences as this would be included in findings.

Is the study design appropriate for the research question?

Yes

Is the rationale for, and objectives of, the study clearly described?

Yes

Are sufficient details of the methods provided to allow replication by others?

Partly

Are the datasets clearly presented in a useable and accessible format?

Not applicable

Reviewer Expertise:

I do research in health service delivery with a focus on models of care that support improved care integration. I have done research in the area of transition from paediatric to adult care for children and youth with complex care needs.

I confirm that I have read this submission and believe that I have an appropriate level of expertise to confirm that it is of an acceptable scientific standard, however I have significant reservations, as outlined above.

# Response

**Published:** 2023-07-05

**Authors:** Melissa Kinch

**Affiliations:** University College Dublin, Ireland

The authors sincerely thank you for taking the time to provide valuable and insightful feedback for the proposed scoping review. We have taken all your suggestions and made the relevant amendments to the scoping review protocol. We are in agreement that your suggestions and feedback have enriched and strengthened the scoping review and express our appreciation. Below, we have provided a response to each piece of constructive feedback in order:

 1. The authors have revised the background section to ensure grammatical errors are corrected and that the appropriate terminology is utilised and is consistent throughout.

 2. As recommended, the second paragraph of the methods section has been revised to include only various multi-disciplinary researchers with expertise in healthcare transition.

 3. The aims and objectives of the scoping review have been revised completely in version two to improve the manageability of the scoping review. The authors recognise that this section was initially very broad and have revised it as recommended.

 4. The authors have decided to only include primary, peer-reviewed research studies so that they can understand how such programmes or interventions were developed or experienced by the end-users, *i.e.*, what specific methodology or methods were used or studies that refer to experiences of named and described interventions. This will help to inform the next phase of the primary researchers overall study. Nonetheless, descriptive studies that provide information on existing programmes or interventions will be included and acknowledged in the scoping review's discussion section.

 The scoping review aims to understand, map, and synthesise what interventions are available to support the healthcare transition. However, the scoping review will also map the qualitative studies, which refer to experiences, barriers, and facilitators of named interventions. 

 5. The aim that was initially proposed surrounding clarifying the definition of transitional care has been removed. The authors recognise that this may be beyond the scope of the review and that it may be more appropriate to undertake a concept analysis to truly understand the concept.

 6. The authors initially utilised the phrase “ *evidence base*” in the review question, as they wanted to ensure that the posed review question appropriately addressed all aims of the review. For example, to ensure that the scoping review question encompassed not only identifying the available interventions but also the studies that refer to experiences of such interventions. Therefore, the authors aim to synthesise all the evidence on the interventions to support the healthcare transition within the context of nephrology services. Nonetheless, the authors have taken your feedback, clarified the research question, and removed the phrase “ *evidence base*”.

 7. The authors recognise the importance of mapping the full extent of literature available for the scoping review. As acknowledged, the primary researcher is bound by time constraints to complete the scoping review promptly as a component of their academic degree. Therefore, the authors have decided to include national grey literature surrounding transitional care, including policies, models of care, *etc.,* within the discussion section of the scoping review to contextualise the interventions that are identified and the experiences of these interventions within the context of the Irish healthcare system where the primary author’s research study into the area will be undertaken.

 8. The authors focus specifically on interventions offered within secondary and tertiary healthcare services, as the primary author’s research study will be conducted in this area. Therefore, the results of the scoping review will be utilised to inform the subsequent phases of the research study. Nonetheless, at least one community-based intervention was recognised from the full-text screening process and will be mentioned in the discussion section of the review.

 9. Though optional in a scoping review, the authors have decided to undertake quality appraisals on the research studies identified. This has been recognised as a step that will enhance the review by providing the reviews readership with the quality of the studies obtained and will ensure that the results of the studies obtained are reliable and trustworthy, as it is proposed that this scoping review will be presented to and discussed with academics and healthcare professionals, young people, and families with experience of healthcare transition. Furthermore, this scoping review will inform the next phase of the research study.

 10. The capitalisation has been removed to ensure consistency in writing. Furthermore, key concepts have been clarified in line with your recommendations.

 11. The screening process has been completely revised to improve clarity. Two reviewers will undertake the complete screening process independently (title and abstract & full text screening). The primary author (MK) will conduct the preliminary data extraction independently due to resource and time constraints to complete the scoping review as part of an academic degree. Nonetheless, this process will be closely supervised and audited by the fourth author (SS), who will supervise, clarify, and agree on the data charting table before sharing the table with all other authors, refining the table based upon their feedback and reaching a consensus on the data that has been charted.

 12. The pilot testing of the literature refers to a pilot testing of the screening process, with the inclusion and exclusion criteria inputted to ensure consistency between reviewers undertaking the screening process. If required, relevant amendments or modifications to the criteria can be made at this stage

 13. A specific list of the data extracted during the data charting process has been included. Minor amendments have been made to the data charting form as the review process has progressed. 

 The authors have included a separate category for experiences in the data chart because some literature that will be included may solely focus on the experiences of an intervention, whereas other papers may describe the intervention and how it was designed but not refer to the experiences of the intervention.

# Reviewer report: Reviewer response for version 1

**DOI:** 10.21956/hrbopenres.14965.r33428

**Published:** 2023-02-22

**Authors:** Kim Mooney

**Affiliations:** 1 School of Nursing, University of Maryland, Baltimore, MD, USA

The goal of this scoping review is to map the evidence for intervention to support AYA with kidney disease from pediatric to adult transition. The authors will use Arskey and O'Malley's approach, which is is a strength, and will seek input from important stakeholders for step 6. Will AYA or their family caregivers provide feedback?

 The introduction was comprehensive. It would help to move the purpose up earlier to guide the reader. Also, there is a notable absence of parent/family caregivers perspectives and acknowledgement of the support they provide across domains for the AYA transition. Are the authors planning to evaluate only AYA-focused interventions or those that incorporate family, as well? This is noted in the inclusion/exclusion criteria, but it would help to make some mention of it in the introduction.

 The databases noted in the quality appraisal section do not match those noted in the search strategy. Even though the preliminary search is the only one noted in the search strategy, this section should reflect the breadth of what is planned. Both sections should match.

 For stage 5, will the authors also collate the demographic data of the studies to account for which areas are represented or not and for which voices are overly-represented or missing? I was not clear if this was to be part of the basic descriptive analysis.

 This is a thoughtful and comprehensive protocol. As noted earlier, there are a few sections that need clarification. I wish the authors well.

Is the study design appropriate for the research question?

Yes

Is the rationale for, and objectives of, the study clearly described?

Yes

Are sufficient details of the methods provided to allow replication by others?

Partly

Are the datasets clearly presented in a useable and accessible format?

Not applicable

Reviewer Expertise:

family, pediatric palliative care, pediatric oncology

I confirm that I have read this submission and believe that I have an appropriate level of expertise to confirm that it is of an acceptable scientific standard.

# Response

**Published:** 2023-07-05

**Authors:** Melissa Kinch

**Affiliations:** University College Dublin, Ireland

The authors would like to thank you for your time providing feedback on our scoping review protocol. We value your feedback and suggestions for improvement and have made all relevant changes as required in the updated version two. We concur that your feedback has greatly enhanced the quality of the scoping review and express our gratitude. We have included all responses to your constructive feedback below, in order:

 1. Step six of this scoping review will seek stakeholder input through an expert reference panel comprising multi-disciplinary healthcare professionals in nephrological transitional care and academia. Furthermore, the authors recognise the importance of involving families and young people experiencing the phenomenon in providing feedback. Therefore, the scoping review results will be presented to PPI groups, comprising AYAs and parents/caregivers, and their feedback will be sought on the interventions sourced.

 2. The introduction has been restructured, with the purpose of the scoping review made explicit earlier. Amendments have been made to the background section, with some content restructured or removed. This decision has been made for the following reasons: 1. To enhance the readability of the review and to ensure that the purpose of the review is not lost, and 2. Congruent with your feedback surrounding the absence of families within this protocol, the authors have provided a space for a dedicated paragraph acknowledging their support during this process.

 3. Though the authors are searching primarily for interventions available to support the healthcare transition for AYAs, the authors agree that healthcare transition does not solely involve or impact upon the young person, but must also support families. Therefore, the authors anticipate that the interventions sourced will also acknowledge and incorporate families. Hence, any intervention that refers to families or familial support or the work that healthcare professionals must do to support the AYAs will be included.

 4. The preliminary search has been revised and repeated using the same databases noted in the search strategy. Both sections now match.

 5. The authors plan on collating the demographic data as described to provide information surrounding areas that are well or under-represented within the literature as part of the descriptive content analysis, using frequency accounts and a narrative account. The "PAGER framework", developed by Bradbury-Jones et al. 2022, has now been included to summarise the results systematically based on patterns, advancements, gaps, evidence for practice, and research recommendations. Areas that are poorly represented could be identified as a gap in the literature and may also provide direction for research recommendations in under-represented areas.

 Reference:

 Caroline Bradbury-Jones, Helen Aveyard, Oliver Rudolf Herber, Louise Isham, Julie Taylor & Lisa O’Malley (2022) Scoping reviews: the PAGER framework for improving the quality of reporting, International Journal of Social Research Methodology, 25:4, 457-470, DOI: 10.1080/13645579.2021.1899596
